# Targeting tumor-associated macrophages in non-small cell lung cancer: mechanisms, prognosis, and therapeutic opportunities

**DOI:** 10.3389/fimmu.2025.1679537

**Published:** 2025-09-22

**Authors:** Dameng Hao, Shengjie Chen

**Affiliations:** 1 Department of Cardiothoracic Surgery, Affiliated Hospital of Jiangsu University, Zhenjiang, China; 2 Medical School of Jiangsu University, Jiangsu University, Zhenjiang, China

**Keywords:** non-small-cell lung cancer, tumor-associated macrophages, tumor microenvironment, metastasis, angiogenesis, therapeutic resistance

## Abstract

Non-small cell lung cancer (NSCLC) remains the most prevalent and lethal form of lung cancer worldwide. Among the diverse components of the tumor microenvironment, tumor-associated macrophages (TAMs) are increasingly recognized as key regulators of NSCLC progression, metastasis, and treatment resistance. TAMs, particularly those polarized toward the M2-like phenotype, facilitate tumor growth through immunosuppression, angiogenesis, epithelial–mesenchymal transition, and extracellular matrix remodeling. They promote immune evasion via PD-L1, IL-10, and TGF-β signaling, and confer chemoresistance through activation of the IL-6/STAT3 and P2X7/STAT6 pathways. Moreover, high infiltration of M2-TAMs and their expression of immune checkpoint ligands have been associated with poor prognosis and, paradoxically, with improved response to PD-1/PD-L1 blockade in certain patients. Emerging therapeutic strategies aim to reprogram TAM phenotypes, inhibit their recruitment, or selectively suppress their immunosuppressive functions. However, challenges such as macrophage plasticity, lack of specific biomarkers, and potential systemic toxicity remain significant barriers. This review provides a comprehensive overview of the biological functions, mechanistic roles, and clinical implications of TAMs in NSCLC, highlighting both their value as prognostic indicators and their potential as therapeutic targets in the era of precision oncology.

## 1 Introduction

Lung cancer persists as the most commonly diagnosed malignancy and remains the foremost cause of cancer-related mortality globally (1). Despite significant advances in molecularly targeted therapies and immunotherapies that have improved clinical outcomes in NSCLC (2, 3), the absence of reliable early screening modalities and the vague nature of initial clinical manifestations contribute to most patients presenting with metastatic disease at diagnosis (4). This underscores an urgent need for innovative therapeutic strategies that can effectively intervene in the complex biological processes underpinning tumor progression.

Compelling evidence has highlighted the tumor microenvironment (TME) as a central driver of cancer development, invasion, and therapeutic resistance (5, 6). The TME encompasses a heterogeneous assemblage of non-malignant stromal and immune cells—including macrophages, endothelial cells, and lymphocytes—interacting within a dynamic network of cytokines and extracellular matrix components that collectively orchestrate tumor behavior (7, 8). Among these, tumor-associated macrophages (TAMs) have emerged as critical regulators of tumorigenesis (9, 10). Induced by tumor-derived cytokines, TAMs adopt an M2-like phenotype and acquire immunosuppressive and pro-tumoral functions, facilitating disease progression across diverse cancer types (11–13). Hence, elucidating the fundamental mechanisms underpinning TAM function is critical for provide promising treatment avenues for patients with NSCLC. This review synthesizes current knowledge on the functional and mechanistic contributions of TAMs in lung cancer, with a particular focus on their implications for NSCLC progression and therapeutic resistance.

## 2 Classification and biological functions of TAMs

Macrophages are multifunctional immune cells with a broad range of physiological roles, including maintaining tissue homeostasis, defending against invading pathogens, and promoting wound healing. Within tumors, most TAMs are enriched at the invasive front and avascular regions of the tumor mass (14, 15). Traditionally, macrophages were thought to be derived primarily from circulating monocytes that migrate into tissues and differentiate. However, recent evidence has demonstrated that a large proportion of tissue-resident macrophages originate from yolk sac progenitors (16, 17). These embryonic precursors undergo local proliferation and differentiation to generate specialized populations such as alveolar macrophages, microglia, and Kupffer cells. In cancer, these resident macrophages, together with recruited monocytes, are activated by diverse signals within the TME, thereby profoundly shaping tumor progression and metastasis (18–20). Increasing evidence underscores the critical role of circulating monocyte recruitment in the establishment of TAM populations (21). During tumorigenesis, inflammatory monocytes from the peripheral blood are attracted to the tumor site by chemokines such as CCL2, as well as cytokines including CSF-1 and VEGF (22–24). Once recruited, these monocytes differentiate into mature macrophages. CCL2, in particular, mediates the recruitment of CCR2-expressing monocytes from the bloodstream into the tumor bed, where they subsequently mature into TAMs (25, 26). Both tumor cells and TAMs amplify CCL2 production, thereby creating a positive feedback loop that further promotes TAM accumulation and proliferation. Tumor growth also promotes the differentiation of CCR2^+^ monocytes into TAMs (27, 28). Additional factors, such as the chemokine CXCL1 and cytokines including platelet-derived growth factor (PDGF) and transforming growth factor-β (TGF-β), also contribute to the polarization of TAMs (29–31).

The TME is characterized by nutrient deprivation, acidosis, and hypoxia, all of which play pivotal roles in regulating TAM polarization and function (32, 33). TAMs display remarkable phenotypic and functional heterogeneity, responding dynamically to contextual signals throughout tumor initiation, progression, and metastasis (34, 35). Conceptually, TAMs are broadly classified into two functional subsets: pro-inflammatory M1 and anti-inflammatory M2 phenotypes (36, 37). M1 TAMs arise in response to IFN-γ and inflammatory cues such as TNF-α, IL-12, and IL-23, and are associated with Th1 immune responses and tumoricidal activity (37–39). These macrophages possess robust antigen-presenting capacity and are marked by CD80, CD86, and CD64 expression (40, 41). In contrast, M2 TAMs are driven by IL-4 and IL-13 signaling, leading to the secretion of IL-10, IL-1 receptor antagonist (IL-1RA), and chemokines that dampen immune activation (42, 43). They express high levels of Arg-1, CD206, and CD163, indicative of an immunosuppressive, pro-tumorigenic phenotype (44). M2 TAMs impair antigen presentation, support Th2 responses, and facilitate tumor progression by promoting metastasis, angiogenesis, and suppression of M1-mediated immunity (36). M2 TAMs drive malignancy progression via three principal mechanisms. First, they facilitate the entry of cancer cells into circulation and promote metastatic spread by activating paracrine signaling pathways (45). Second, these macrophages release a spectrum of immunoregulatory factors such as TGF-β, IL-10, Arg-1, and nitric oxide (NO) which sustain an immunosuppressive microenvironment conducive to tumor expansion (46). Third, they potentiate neovascularization, thereby enabling tumor proliferation and aiding tissue regeneration in the aftermath of oncologic therapies (47).

## 3 Mechanistic roles of TAMs in NSCLC

Within the dynamic tumor microenvironment, TAMs orchestrate a spectrum of oncogenic programs, including tumor cell proliferation (31), angiogenesis (48), drug resistance (49), and immune escape (50), through highly coordinated molecular circuits that collectively sustain NSCLC progression.

### 3.1 TAMs in the proliferation, invasion, and metastasis of NSCLC

Tumor recurrence and metastasis remain the predominant drivers of mortality in NSCLC, with TAMs emerging as pivotal regulators of these processes through multifaceted interactions with malignant and stromal components (51). Tumor cells secrete diverse chemokines that recruit macrophages and other inflammatory cells into the tumor stroma, where TAMs, in turn, release growth factors, cytokines, chemokines, and mediators such as VEGF, PDGF, IL-10, CXCLs, EGFR ligands, and FGFs. These substances exert direct mitogenic effects and stimulate angiogenesis, collectively enhancing NSCLC growth and dissemination (52, 53). TAM-derived epidermal growth factor (EGF) drives the formation of elongated tumor cell protrusions that augment invasion, reinforced by a CSF-1/EGF positive feedback loop that markedly amplifies metastatic behavior (54). Inflammatory mediators from TAMs activate NF-κB and STAT3, further sustaining tumor cell proliferation and survival (55). EGFR ligands are particularly relevant in NSCLC, where receptor dimerization triggers potent proliferative cascades. Notably, NOX4-driven M2-polarized macrophages exhibit elevated JNK activity and secrete heparin-binding EGF-like growth factor (Hb-EGF), thereby stimulating NSCLC proliferation, identifying TAMs as a key EGF source in the tumor microenvironment (56, 57).

TAM polarization toward the M2 phenotype, driven by IL-4 or IL-13, dampens anti-tumor T cell responses and enhances tumor-promoting processes including angiogenesis, proliferation, and invasion (58). Through the TLR4/IL-10 axis and secretion of TGF-β, a master regulator of EMT, TAMs activate TGF-β/β-catenin signaling and upregulate SOX9, which facilitates NSCLC cell migration and invasion (59, 60). SOX9, in turn, orchestrates cytoskeletal reprogramming by promoting mesenchymal markers such as vimentin and fibronectin while repressing epithelial proteins including E-cadherin (61–63), leading to loss of polarity and increased cellular motility. Furthermore, TAMs secrete a variety of factors, including MMP-9, VEGF, COX-2, and urokinase plasminogen activator, which remodel the extracellular matrix and promote invasion (64). Microfluidic modeling of the tumor ecosystem has revealed that M2 macrophages elevate CRYAB expression in lung cancer cells, driving EMT and metastasis (65). TAMs also facilitate Ezrin phosphorylation-mediated EMT in lung adenocarcinoma via FUT4-dependent fucosylation and synthesis of the LeY antigen (66). Phosphorylated Ezrin acts as a linker between the plasma membrane and actin cytoskeleton, promoting the formation of invadopodia and lamellipodia, which are essential for directional cell migration and metastasis. This reorganization of the actin cytoskeleton supports enhanced invasive behavior of NSCLC cells (67). Moreover, Oct4 upregulation in lung cancer cells induces M2 polarization of macrophages via M-CSF overexpression, establishing a pro-tumorigenic Oct4/M-CSF axis (68). Other regulatory pathways include the GNASAS1/miR-4319/NECAB3 axis, which modulates macrophage polarization to favor NSCLC progression (69), and the SR-A1/MAPK/IκB/NF-κB signaling cascade, where SR-A1 deficiency in TAMs leads to SAA1 upregulation, enhancing both macrophage migration and tumor invasion (70). Besides, Li et al. (71) identified the C-type lectin receptor Mincle as a critical immunosuppressive factor in TAMs. Mincle activation promotes M2 polarization and tumorigenesis via the Syk/NF-κB axis, thereby representing a potential target for immunotherapy. Taken together, these findings underscore TAMs as central orchestrators of NSCLC progression, facilitating tumor growth and dissemination through reciprocal tumor–macrophage interactions, pro-oncogenic signaling, and dynamic reprogramming of the tumor microenvironment.

### 3.2 TAMs promote angiogenesis in NSCLC

TAMs are central regulators of tumor angiogenesis, a prerequisite for tumor growth and invasion (72). By supporting oxygen and nutrient supply and facilitating metabolic waste removal, TAM-induced neovascularization sustains tumor expansion. This process is orchestrated through the secretion of pro-angiogenic mediators and active remodeling of the TME (73). Key angiogenic effectors released by TAMs include VEGF, TNF-α, IL-1β, IL-8, PDGF, basic fibroblast growth factor (bFGF), thymidine phosphorylase, and matrix metalloproteinases (MMPs), establishing a direct link between TAM function and intratumoral vascularization (48). Notably, *in vitro* studies show that M2-polarized macrophages elevate VEGF levels and enhance angiogenic responses (74). Furthermore, bidirectional crosstalk between TAMs and NSCLC cells via placental growth factor (PLGF)/Flt-1 and TGF-β signaling intensifies vascular sprouting and tumor progression (75). TAM-derived osteopontin (OPN) promotes cyclooxygenase-2–dependent PGE2 production and MMP-9 expression, facilitating vascular remodeling and metastatic dissemination (76). Hypoxia within the TME acts as a dominant driver of TAM-mediated angiogenesis (77). Under hypoxic stress, hypoxia-inducible factors HIF-1α and HIF-2α are upregulated in TAMs, transcriptionally activating VEGF and PLGF expression and reinforcing pro-angiogenic signaling in NSCLC (78–80). HIF-1α also enhances glycolytic metabolism and TIE2 expression in a subset of pro-angiogenic TAMs known as TIE2^+^ TAMs (81, 82). These cells are enriched in perivascular hypoxic regions and promote angiogenesis via the ANG2/TIE2 signaling axis (83). This pathway not only facilitates endothelial sprouting and vascular stabilization but also recruits additional TAMs to the angiogenic niche, forming a feed-forward loop that sustains tumor vascularization (84).

**Figure 1:**
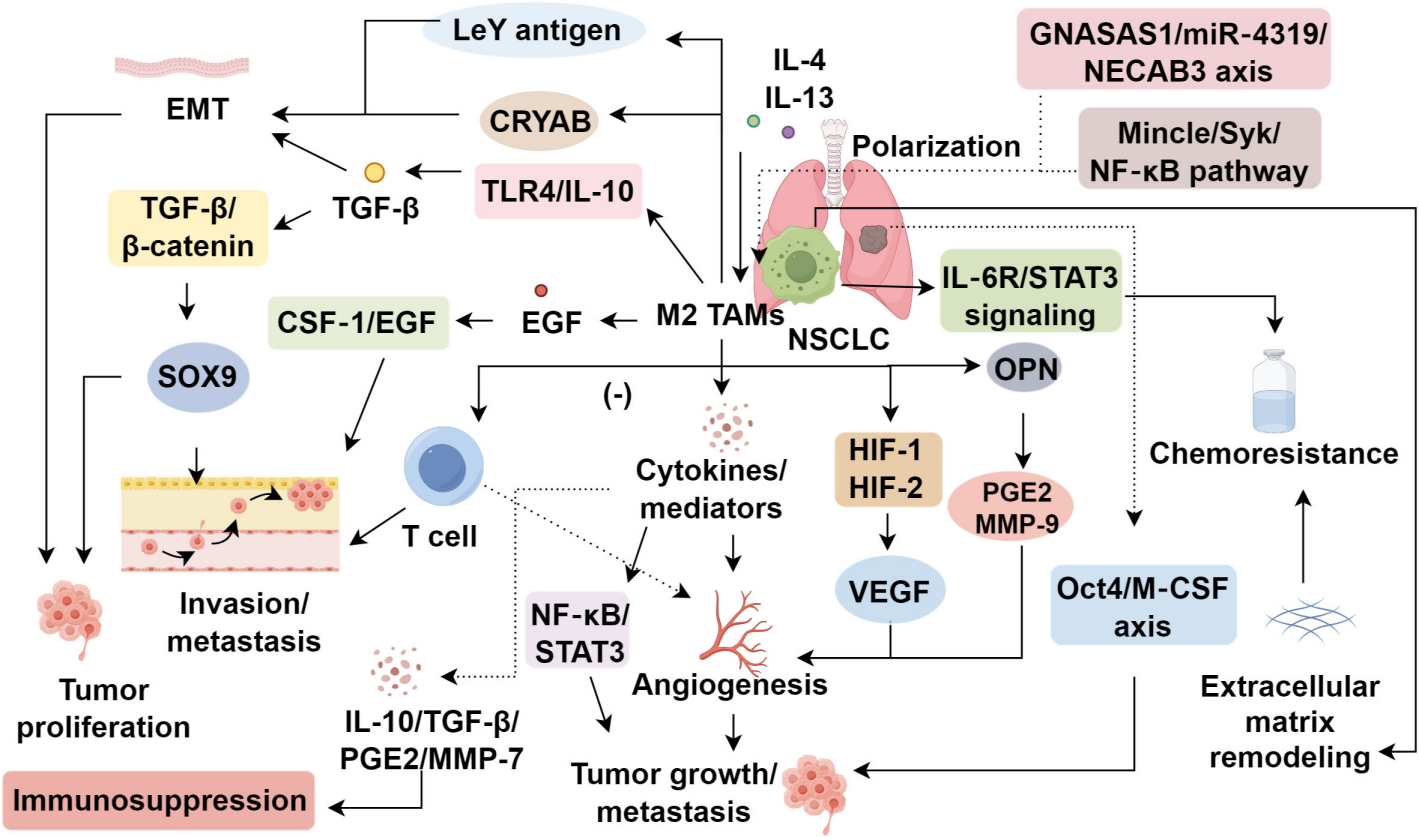
Roles of tumor-associated macrophages in non-small cell lung cancer progression.

Beyond soluble factors, TAM-derived exosomes contribute to angiogenesis by transferring miR-155-5p and miR-221-5p to endothelial cells, thereby stimulating tumor-associated neovascularization (85). Within hypoxic domains, upregulation of HIFs reinforces the expression of VEGF, PLGF, and ANG2, while heightened TIE2 expression in TAMs amplifies vascular signaling cascades (86). Pharmacological interventions such as ginsenoside-Rh2 (G-Rh2) can reprogram TAM polarization from the pro-tumoral M2 phenotype toward the M1 phenotype, suppressing tumor cell migration and downregulating angiogenic factor expression (87). In NSCLC, M2-type TAMs are implicated in both angiogenesis and lymphangiogenesis by inducing VEGF-A and VEGF-C in tumor cells. Immunohistochemical analyses reveal that stromal expression of CD68 and CD163 positively correlates with VEGF-A/C levels, and the abundance of CD163^+^/CD68^+^ TAMs is significantly associated with poor prognosis in NSCLC patients (37, 48). Collectively, Collectively, these findings identify TAMs as critical enablers of tumor vascularization and present a compelling rationale for therapeutically targeting their angiogenic and lymphangiogenic functions in NSCLC (**Figure 1**).

### 3.3 TAMs promote drug resistance in NSCLC

One of the major challenges in NSCLC therapy is the development of resistance to chemotherapy and targeted agents (88). TAMs play a pivotal role in this process by promoting tumor growth, survival, and resistance mechanisms (89). Preclinical models have demonstrated that TAMs secrete IL-6, which drives chemoresistance through IL-6R/STAT3 signaling; for example, in colorectal cancer, TAM-derived IL-6 confers resistance to 5-fluorouracil (5-FU) (90). Similarly, Similarly, exposure to chemotherapeutic agents such as cyclophosphamide, paclitaxel, and doxorubicin in murine models of lung cancer promotes CD206^+^ TAM expansion, which facilitates tumor revascularization and relapse (91). In lung cancer, chemotherapy-induced IL-34 further strengthens TAM-mediated resistance (92). Beyond cytokine release, TAM-driven extracellular matrix remodeling alters tumor–macrophage interactions and reduces tumor sensitivity to chemotherapy and radiotherapy (93, 94).

Clinically, M2-polarized TAMs have been associated with poor prognosis due to their capacity to secrete growth factors and inhibit apoptotic pathways, thereby reducing tumor susceptibility to cytotoxic therapy (95). *In vitro*, cisplatin-resistant NSCLC cell lines (A549R, H460R) exhibit elevated self-renewal capacity and release macrophage migration inhibitory factor (MIF), which skews macrophages toward an M2 phenotype and fosters metastatic progression (96). Zhang et al. revealed M2 TAM infiltration predicts post-chemotherapy recurrence and lymph node metastasis, suggesting their potential as early imaging biomarkers (97). More recently, P2X7 signaling has emerged as a key driver of TAM-mediated immunosuppression and therapeutic resistance (98). Engagement of P2X7 on macrophages activates the STAT6/IRF4 transcriptional axis, reinforcing M2 polarization and enhancing the secretion of immunosuppressive mediators including IL-10, arginase-1, and TGF-β. This environment not only supports tumor progression but also directly impairs CD8^+^ T-cell effector functions and contributes to T-cell exhaustion, thereby reducing the efficacy of PD-1/PD-L1 immune checkpoint blockade (99). Inhibition or genetic deletion of P2X7 reverses this polarization, enhances inflammatory gene expression, and restores checkpoint inhibitor responsiveness in NSCLC models, highlighting the P2X7/STAT6 pathway as a mechanistic barrier to successful immunotherapy (100).

### 3.4 TAMs in shaping the immunosuppressive microenvironment of NSCLC

TAMs constitute a dominant immune cell population within the TME and play a pivotal role in coordinating innate and adaptive immunity. In NSCLC, TAMs are predominantly polarized toward an M2-like phenotype, which orchestrates immunosuppression via the secretion of cytokines, chemokines, and metabolic enzymes that collectively attenuate antigen presentation and inhibit effector T-cell responses (101, 102). These macrophages release IL-10, TGF-β, PGE2, and MMP-7, fostering a tolerogenic niche that impairs cytotoxic lymphocyte recognition and elimination of malignant cells (103). TGF-β is a key orchestrator of this suppressive environment; it dampens NK cell cytotoxicity, impedes dendritic cell (DC) migration, promotes Th2 differentiation, and transcriptionally represses cytotoxic mediators such as granzyme A/B, IFN-γ, and FasL (104–107). Moreover, TGF-β facilitates Treg induction and recruitment, further amplifying immune suppression (108), and synergistically enhances IL-10 production, thus skewing Th1/Th2 balance toward Th2 dominance (109). IL-10 inhibits NF-κB signaling and curtails the secretion of pro-inflammatory cytokines (TNF-α, IL-6, IL-12) and IFN-γ, expediting immune escape (110–112). Simultaneously, TAM-expressed arginase 1 depletes extracellular L-arginine, curbing T-cell proliferation and downregulating TCR expression (113, 114). The combined effects of IL-10, TNF-α, and IFN-γ further induce B7-H4 on tumor cells, thereby promoting T-cell apoptosis and impeding cytotoxic lymphocyte-mediated tumor eradication (115, 116).

In parallel, immune checkpoint ligands such as PD-L1, PD-L2, CD86, and CD80 expressed by TAMs engage PD-1 and CTLA-4 on T cells, culminating in CD8^+^ T-cell exhaustion (117, 118). TAM-derived TGF-β and PGE2 additionally inhibit DC maturation, thereby disrupting the interconnectivity of innate and adaptive responses (119, 120). Exosomes released by TAMs reprogram immature DCs toward tolerogenic phenotypes, further crippling antitumor immunity (121, 122). Within the NSCLC TME, Tøndell et al. uncovered elevated CD200R1/CD200 signaling between TAMs and T cells, as well as enhanced LILRB expression on M2-TAMs, nominating these as potential immunotherapeutic targets (123). Studies have revealed that PI3Kγ functions as a critical switch regulating TAM phenotype: its activation promotes Akt–mTOR signaling that represses NF-κB but drives C/EBPβ-mediated transcription favoring immune suppression; conversely, PI3Kγ inhibition reactivates NF-κB–driven proinflammatory genes and restores CD8^+^ T-cell cytotoxicity (124). Reprogramming strategies also include targeting the scavenger receptor MARCO or its ligand IL-37/IL-37R, which La Fleur et al. demonstrated reinstates T and NK cell function, curtails Treg activity, and enhances antitumor responses (125). Additionally, EGFR–AKT/ERK1/2 signaling has been shown to upregulate ILT4 in NSCLC cells, facilitating M2-TAM recruitment and dampening T-cell immunity; blocking ILT4 synergizes with PD-L1 inhibitors in EGFR wild-type, but not EGFR-mutant tumors—underscoring an EGFR-driven immune evasion mechanism (126).

## 4 Prognostic impact of TAMs in NSCLC

The heterogeneity of M2-polarized TAMs confers distinct prognostic implications across human malignancies (78, 127). In a study of 509 NSCLC specimens, Li et al. (128) demonstrated a positive correlation between TAM-derived osteopontin (TOPN) and PD-L1 expression within the tumor microenvironment. Both TOPN and PD-L1 were identified as independent prognostic factors for overall survival and disease-free survival in NSCLC patients. Mechanistically, TOPN upregulates PD-L1 expression in NSCLC cells via activation of the NF-κB signaling pathway, and *in vivo* models confirmed that TOPN-induced PD-L1 facilitates tumor progression (128). In a parallel cohort of approximately 500 NSCLC patients, Liu et al. (129) found that TAMs represent the dominant subset of PD-L1–expressing immune infiltrates. PD-L1 expression on TAMs exhibited a strong positive correlation with both tumor cell PD-L1 levels and the extent of CD8^+^ T-cell infiltration. Strikingly, in patients undergoing anti–PD-1 therapy, high TAM-derived PD-L1 expression predicted improved overall survival (129), implying a potential predictive utility for immunotherapy responsiveness. Similarly, Gross et al. reported that PD-L1 expression on either TAMs or tumor cells was linked to improved survival outcomes in patients receiving adjuvant chemotherapy (130). However, PD-L1 expression on TAMs does not uniformly correlate with better prognosis across all settings. In the absence of immunotherapy, elevated PD-L1 on TAMs may also reflect a highly immunosuppressive TME, which contributes to poor tumor control, highlighting its potential prognostic ambiguity (131, 132). These findings provide a rationale for prospective studies and the development of chemo-immunotherapeutic strategies in lung cancer. The immunological landscape further complicates prognostication. Numerous studies indicate that M2-like TAMs often constitute over 80% of the macrophage compartment in NSCLC TMEs and are broadly linked to adverse clinical outcomes. Accordingly, routine evaluation of PD-L1 expression on TAMs serves not only as a prognostic biomarker but also as a predictive indicator for responsiveness to PD-1 blockade therapies in NSCLC (133).

## 5 Conclusion

Tumor-associated macrophages (TAMs) are deeply implicated in the pathogenesis and progression of NSCLC, contributing to tumor proliferation, angiogenesis, immune evasion, and resistance to therapy. The predominance of M2-like TAMs within the tumor microenvironment supports oncogenic signaling via secretion of VEGF, TGF-β, and IL-10, promotes epithelial–mesenchymal transition and metastasis, and suppresses cytotoxic immune responses through PD-L1 expression and arginase-1–mediated T-cell dysfunction. Mounting evidence also highlights their prognostic value, with high TAM density and PD-L1 expression correlating with disease progression and, paradoxically, response to immunotherapy in select contexts.

Despite emerging strategies to target TAMs such as reprogramming M2 to M1 phenotypes, disrupting recruitment signals, or inhibiting immunosuppressive mediators, several challenges remain. TAMs exhibit profound plasticity, dynamically adapting to environmental cues, which complicates durable therapeutic intervention. The lack of specific biomarkers to distinguish functional TAM subsets hinders precision targeting, while broad depletion strategies risk impairing normal tissue immunity. Future studies should integrate single-cell profiling and spatial transcriptomics to decode TAM heterogeneity, and prioritize the development of biomarker-driven combinatorial approaches that safely and effectively reshape the immunological landscape of NSCLC.
